# Experiences, outcomes and unmet needs of caregivers of children with Cerebral Palsy in Spain: Protocol for a mixed-methods study

**DOI:** 10.1371/journal.pone.0342763

**Published:** 2026-03-13

**Authors:** Clàudia Arumí-Trujillo, Francisco José Verdejo-Amengual, Oriol Martínez-Navarro, Jord J.T. Vink, Fran Valenzuela-Pascual

**Affiliations:** 1 Faculty of Nursing and Physiotherapy, Universidad de Lleida, Lleida, España; 2 Translational Neuroimaging Group, Center for Image Sciences, University Medical Center Utrecht and Utrecht University, the Netherlands; 3 Group of Studies on Society, Health, Education and Culture (GESEC), Universidad de Lleida, Lleida, España; 4 Research Group of Health Care (GReCS), Lleida Biomedical Research Institute’s Dr. Pifarre Foundation (IRB Lleida), Lleida, España; 5 Center of Excellence in Rehabilitation Medicine, University Medical Center Utrecht Brain Center, University Medical Center Utrecht, Utrecht University and De Hoogstraat Rehabilitation, the Netherlands; Federal University of Paraiba, BRAZIL

## Abstract

Cerebral Palsy is one of the most prevalent motor disabilities in childhood, significantly impacting both children and their caregivers. This mixed-methods study explores the experiences, psychological well-being, and unmet needs of caregivers of children with Cerebral Palsy. Using an explanatory sequential design (QUAN → QUAL), first it will be assessed burden, stress levels, and quality of life of caregivers through standardized questionnaires (PedsQL-FIM, ZBI, PSS-14). In the second phase, semi-structured interviews will be conducted to provide a deeper understanding of these variables. Other studies indicate that caregivers experience heightened stress and decreased quality of life, influenced by their child’s functional limitations and the lack of adequate social and healthcare support. Moreover, many caregivers struggle with navigating medical systems, balancing personal and professional responsibilities, and managing emotional distress. This study will highlight the urgent need for family-centered interventions, psychosocial support, and healthcare policies that address not only the medical needs of children with Cerebral Palsy but also the well-being of their caregivers. By integrating quantitative and qualitative data, this research will provide comprehensive insights into the caregiving burden and will offer recommendations for improving caregiver support strategies. The Clinical Trial is registered at ClinicalTrials.org with the registration number NCT06912373.

## Introduction

Neurological disorders are characterized by impairments affecting various regions of the brain or nervous system and requires comprehensive, multidisciplinary approach to their management, particularly in paediatric populations [1,2]. Effective care for children with such conditions relies on a thorough understanding by healthcare professionals, caregivers, and parents [2,3].

Parents of children with developmental disabilities face substantial challenges in their daily lives, as they must respond to their child’s complex needs while simultaneously fulfilling their parental role. This responsibility demands substantial time, physical effort, and emotional investment [4]. Caregiving demands may adversely affect parents’ physical and psychological health and place strain on overall family functioning, partly through their influence on stress management, self-perception, and perceived social support [5,6].

The birth of a child with Cerebral Palsy often necessitates significant adjustments to family routines and may tigger emotional responses in parents, including denial, grief, and guilt. These reactions are closely related to the process of adapting to the child’s functional limitations and the long-term caregiving demand associated with the condition [7,8].

Elevated levels of parenting stress have been associated with impaired parenting practices, disruptions in parent-child relationships, and adverse outcomes for the children’s health and development [9,10]. Early identification and intervention targeting parenting stress—through family-centred care approaches—are therefore essential to support both child development and family well-being [11,12]. Moreover, higher levels of perceived stress have been linked to increased anxiety and reduced quality of life among caregivers [13].

Although qualitative research has explored on caregiving’ experiences across different populations, to the best of the authors’ knowledge, there is no qualitative study that explores the emotional experiences, stress management, challenges, and unmet needs of caregivers of children with motor/physical disabilities. Moreover, caregiving experiences and available support may vary substantially across healthcare systems and cultural contexts.

In this context, the present study aims to comprehensively investigate the experiences, psychological well-being, and unmet needs of caregivers of children with Cerebral Palsy in Spain through a sequential mixed-methods design. The study will fill an important gap in literature, providing context-specific evidence that can guide healthcare providers and policymakers in developing interventions and support strategies tailored to caregivers’ realities.

The Clinical Trial is registered at ClinicalTrials.org with the registration number NCT06912373, in April 2025, named “Experiences of Caregivers of Children with Cerebral Palsy (https://clinicaltrials.gov/study/NCT06912373?id=NCT06912373&rank=1)

## Materials and methods

To address the research question, the authors will use an **explanatory sequential mixed-methods** design consisting of three phases **(QUAN → Connection ◊ QUAL)**. The rationale for choosing this approach is that the analysis of the quantitative data will provide a general understanding of burden, stress and quality of life among caregivers of children with Cerebral Palsy. The subsequent analysis of the qualitative data will further explain the statistical results through an in-depth exploration of the experiences and needs of the participants [14].

A three-phase procedure will be used:

- QUANT phase: Quantitative data will be collected and analysed using three questionnaires, providing the authors with a comprehensive profile of participants’ stress levels, quality of life, and caregiver burden. This data will also enable the identification of specific subgroups that will be the primary focus of the subsequent qualitative phase, while informing the development of new research questions for a deeper exploration during this phase.
- Connection phase: Firstly, based on the categories identified through the quantitative questionnaires, the content of the semi-structured interviews will be developed. Secondly, the results from the quantitative phase will inform the planning of the qualitative phase, including the selection of a potential subsample and the development of new questions that cannot be addressed through quantitative data.
- QUAL phase: semi-structured personal interviews, based on the results of the quantitative data and followed by thematic analysis.

This study adopts an explanatory sequential mixed-methods design because the quantitative phase and the qualitative phase address complementary aims that cannot be achieved by either approach alone. The quantitative phase provides a population-level profile of caregiver burden, perceived stress, and quality of life, and examines associations among variables (e.g., sociodemographic factors, caregiving characteristics, and PedsQL-FIM subscales). These results will guide the development of the interview guide for the qualitative phase. In turn, the qualitative phase will generate in-depth explanations—mechanisms, contexts, and meanings—behind statistical patterns, allowing us to produce integrated, practice-relevant inferences. Integration will occur at three points; i) The results from the quantitative phase will help to plan the qualitative phase, including formulation of new questions ii) The development of the qualitative interview guide will be based on the categories identified in the questionnaires iii) The qualitative data will be integrated with the quantitative data to obtain a more comprehensive understanding of the quantitative results.

### Hypothesis and study objectives:.

Based on previous research on caregiving for children with Cerebral Palsy, we hypothesize that: (1) higher caregiver stress and burden will be associated with lower quality of life; (2) higher educational and income levels will be associated with lower burden and higher quality of life; and (3) the type or severity of Cerebral Palsy will show weaker associations with caregiver outcomes than psychosocial and contextual factors. These hypotheses are grounded in existing literature and will be examined in the regression models and subsequently elaborated in the qualitative phase, following the explanatory sequential mixed-methods design. Therefore, these hypotheses are considered hypothesis-generating and explanatory and will be further explored and contextualized through the qualitative phase. The overarching objective of this study is to generate an integrated understanding of the experiences, outcomes, and unmet needs of caregivers of children with Cerebral Palsy in Spain. Particularly, the quantitative analysis of caregiver burden, stress, and quality of life will provide the first objective; give a general profile, which will then inform and modify the development of the qualitative interview guide. The qualitative phase, second objective, will explore caregivers lived experiences, coping strategies, and perceived needs in depth, thereby expanding upon and contextualizing the quantitative findings. Stress, caregiver burden, and quality of life are conceptualized as co-primary constructs of interest, reflecting complementary and interconnected dimensions of the caregiving experience.

### Recruitment and data collection

#### Quantitative phase.

Before the recruitment begins, the principal investigator will present the project to the paediatric service of the Arnau de Vilanova University Hospital of Lleida (HUAV). During medical consultations, the paediatric service will inform eligible participants about the study. If the caregiver agrees to participate, they will give consent for the paediatric service to share their contact details with the research team. The investigators will then contact the participant to explain the project in detail, schedule a first meeting to sign the Informed Consent, and complete the initial questionnaires. Additionally, participants who sign the informed consent will be asked to provide their contact information for potential follow-up regarding the qualitative interview.

In the first meeting, participants who meet the inclusion criteria and have signed the informed consent will be directed to a study homepage hosted on the university’s website. This website will provide a link to a secure platform specifically designed for collecting research data (PedsQL-FIM, ZBI, PSS-14).

#### Sociodemographic data.

Participants’ sociodemographic data will include descriptive information such as age, sex, household income, among others.

#### Inclusion criteria.

- >18 years old
- Being a caregiver of a child with Cerebral Palsy
- Spanish/Catalan speaking
- Willing to talk about their experiences and be audio/video-recorded.
- Accept and sign the informed consent form

#### Qualitative phase.

The interview guide will be refined based on the results of the quantitative phase. Additionally, the findings from the quantitative questionnaires will inform the selection of participants for the interview phase. The interview will take place in the Faculty of Nursing and Physiotherapy of the University of Lleida. Interviews will be conducted in either Spanish or Catalan (participants’ choice) and will be recorded in audio or video, with participants’ previous consent. Once all questionnaire data has been analysed, a subgroup will be created based on the results obtained, which will be related to the categories identified in the PedsQL™ Family Impact Module [15] and the Zarit Burden Interview [16], both in their Spanish versions.

#### Sample size considerations.

The quantitative sample size was estimated using GRANMO (estimation of a population mean). Assuming a 95% confidence level, a standard deviation of 21, based on Spanish normative data for the PedsQL-Family Impact Module [17]. Assuming an accessible finite hospital population of approximately 40 eligible caregivers and a precision of ±5 points on the 0–100 scale; the required sample is n = 26 completers. Allowing for an anticipated 15% attrition rate, the recruitment target is approximately 31 caregivers. This sample ensures estimation of the mean PedsQL-FIM score with adequate precision for the finite population. In addition, with n = 25–30 completers, two-tailed tests at α = 0.05 will have approximately 80% power to detect correlations of r = 0.50–0.54, providing sufficient power for moderate-to-large associations while acknowledging that smaller effects may be underpowered

#### Study status and timeline.

**Fig 1:**
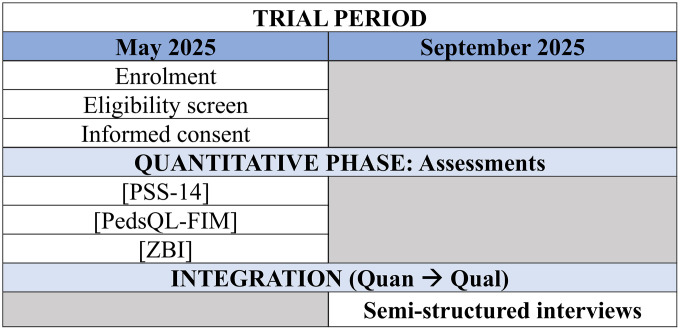
Participant timeline: Schedule of enrollment, interventions and assesments. PSS-14: Perceived Stress Scale, PedsQL-FIM: Qualoty of Life by PedsQL^TM^ Family Impact Module, ZBI: Zarit Burden Interview.

At the time of manuscript submission, the study has not yet started. The start date of the recruitment period for this study is expected to begin 02/06/2025 and expected to be completed by end of June 2025. Data collection is anticipated to be finalized by August 2025, and preliminary results are expected to be available by September 2025. This timeline is subject to minor adjustments depending on recruitment rates and operational logistics S1 Fig (Fig 1.)

### Measures

#### Perceived Stress Scale (PSS-14).

The PSS-14 is short and easy adaptation of the original Perceived Stress Scale (PSS), with the original author being Cohen, S. The European Spanish adaptation was conducted by Remor, E. [18].

The PSS measures the degree to which individuals perceive life situations as stressful over the past month, focusing on subjective control over unpredictable events and the distress associated with perceived lack of control. The PSS is more closely related to subjective evaluations of life events than objective measures of stressors [19]. The 14-item European Spanish version of the Perceived Stress Scale (PSS-14) will be employed to assess caregivers’ levels of perceived stress. This instrument comprises 14 items, each rated on a five-point Likert scale ranging from 0 to 4 (0 = never, 1 = rarely, 2 = sometimes, 3 = usually, 4 = almost always), yielding a total score between 0 and 56. Higher scores indicate greater perceived stress. The European Spanish adaptation of the PSS-14 has demonstrated acceptable psychometric properties, including good internal consistency (Cronbach’s alpha = .81), satisfactory test-retest reliability (r = .73), and evidence of concurrent validity and sensitivity [20].

#### Quality of Life by PedsQL™ Family Impact Module (PedsQL-FIM).

To assess the impact of paediatric chronic health conditions on caregivers and the family, the Spanish version of the PedsQL™ Family Impact Module will be used [15,21]. This module employs a multidimensional parent self-report to evaluate physical, emotional, social, and cognitive functioning, as well as communication and worry. The module can be completed within 5–10 minutes [21,22] The Pediatric Quality of Life Inventory Family Impact Module (PedsQL-FIM) will be utilized to evaluate the impact of paediatric health conditions on caregiver and family functioning. This instrument consists of 36 items, demonstrating excellent internal consistency reliability (Cronbach’s α = 0.97) (21). It measures eight core dimensions: physical functioning (6 items), emotional functioning (5 items), social functioning (4 items), cognitive functioning (4 items), communication (3 items), worry (5 items), daily activities (3 items), and family relationships (5 items). Responses are rated on a 5-point Likert scale (0 = never a problem to 4 = almost always a problem), then reverse scored and linearly transformed to a 0–100 scale (0 = 100, 1 = 75, 2 = 50, 3 = 25, 4 = 0), with higher scores indicating better perceived functioning. The total score is calculated by summing all item scores and dividing by the number of items completed [23].

#### Zarit Burden Interview (ZBI).

The Zarit Burden Interview is a widely used self-report instrument for measuring caregiver burden. ZBI assesses caregivers´ perceptions of burden that may inadvertently affect their health, personal, social or financial wellbeing [16].

Caregivers are requested to assess the level of burden they experience while caring for a loved one. Burden is defined as the degree to which a caregiver perceives negative effects on their emotional and physical health, social life, and finances, which may hinder their ability to provide care. Responses vary from “not at all” to “extremely.” It consists of 22 items, with the total score calculated by summing the scores of all the indicated items. It is suggested that a score from 0 to 21 means no to mild burden, 21–40 indicates mild to moderate burden, 41–60 indicates moderate to severe burden, and more than 61 means severe burden [24].

The Spanish version developed by Martin-Carrasco et al. [25] showed a good internal reliability (Cronbach alpha coefficient of 0.92) [17,26].

### Data analysis

#### Quantitative data.

The quantitative data will be analysed using descriptive statistics and frequency counts. Sociodemographic characteristics will be described. Descriptive statistics will be reported with means and standard deviations for continuous variables with normal distribution (age, child’s age, PSS-14, PedsQL-FIM, ZBI), or medians and interquartile ranges for variables without normal distribution. For categorical variables (sex, educational level, severity), both absolute and relative frequencies will be presented.

For the inferential analyses, Spearman’s correlations will be used to explore relationships among questionnaires (stress, burden, and quality of life) as well as to correlate caregiver outcomes (stress, burden, quality of life) across subgroups defined by sociodemographic variables (e.g., economic status, education level), child-related variables (e.g., severity of Cerebral Palsy, mobility), and caregiving factors (e.g., relation with the child). Primary analyses will use multivariable linear regression to model each caregiver outcome: (i) perceived stress (PSS-14), (ii) caregiver burden (ZBI), and (iii) quality of life (PedsQL-FIM total). Separate regression models are specified for stress, caregiver burden, and quality of life, consistent with their conceptualization as co-primary constructs, each examined to characterize different but interrelated dimensions of caregiver outcomes. Given the finite population constraints (approximately 40 eligible caregivers)), each model will include only the most relevant predictors (up to 2–3 per model), carefully selected on priori literature and clinical plausibility (e.g., socioeconomic status, caregiving duration, and child functional severity/mobility). This parsimonious approach reduces multiple comparisons and allows adjustment for potential confounders. Model assumptions, including linearity, normality of residuals, and homoscedasticity, will be evaluated using standard diagnostics such as residual plots and Shapiro-Wilk tests. If model assumptions are violated, data transformations or alternative statistical techniques will be considered. Results will be reported as standardized regression coefficients (β) with 95% confidence intervals. Analyses will be conducted using IBM SPSS Statistics v28 (or equivalent software) with significance level of α = 0.05 (two-tailed).

#### Qualitative data.

Interviews will be audio- or video-recorded, transcribed verbatim, and analysed using a thematic analysis approach. Both deductive coding (guided by the quantitative findings and existing literature) and inductive coding (to capture emerging themes) will be applied. Codes will be organized into categories and higher-level themes to reflect caregivers lived experiences, coping strategies, and unmet needs. Two researchers will independently code a subset of transcripts to ensure inter-coder reliability, and discrepancies will be resolved through discussion until consensus is reached. Triangulation across researchers will further enhance validity. ATLAS.ti software will be used to manage and code the data [27,28]. It is anticipated a qualitative sample of approximately 10–15 participants, although data collection will continue until saturation is reached, defined as the point where no new relevant themes emerge. The approach will follow the Consolidated Criteria for Reporting Qualitative Research. This approach allows for analysis of the data with a set of expected themes informed by previous knowledge or research [29]. Integration of qualitative findings with quantitative results will be achieved through joint displays, side-by-side comparisons, and narrative weaving, allowing a comprehensive interpretation of caregiver outcomes and experiences. The first author will handle the interview analysis, while a different researcher will be involved in information triangulation to ensure the validity and reliability of the materials [30]. Subsequently, all authors will contribute to interpreting the data.

### Integration of quantitative and qualitative data

Integration will occur at several stages of the study. During the *connection phase*, quantitative findings will guide the development of the qualitative interview guide and the selection of participants for in-depth interviews. At the *interpretation and reporting stage*, integration will be achieved using joint displays, side-by-side comparisons, and narrative weaving to connect statistical results with qualitative themes. Joint displays will visually align quantitative patterns (e.g., caregiver burden, stress, and quality of life scores) with representative qualitative excerpts to illustrate convergence or divergence between the datasets. Side-by-side comparisons will be used in the Results section to present findings from both phases within the same thematic structure, while narrative weaving will be employed in the Discussion to synthesize and interpret the integrated evidence. This multi-level integration approach will provide a comprehensive and coherent understanding of caregivers’ experiences and outcomes.

#### Dissemination plans.

The results of the study will be communicated to participants, healthcare professionals, and the broader public through several channels. Findings will be reported in the corresponding clinical trial registry, submitted for publication in peer-reviewed journals, and presented at relevant scientific conferences. A plain language summary will be prepared and made available to participants upon request. Additionally, healthcare providers involved in the study will receive a summary of the main outcomes to support the translation of findings into practice. Upon completion of the study, all anonymized quantitative datasets (including summary scores and data points underlying statistical analyses) and de-identified coded qualitative transcripts will be deposited in Zenodo (DOI 10.5281/zenodo.18435559) and made publicly available without restriction. Quantitative data will be shared in CSV format, and qualitative data in PDF/TXT format. To ensure participant confidentiality, all personal identifiers will be removed prior to data sharing. Any additional supporting information will be provided as supplementary files at the time of publication.

### Ethical issues

The study follows the Declaration of Helsinki and the “Guidelines for Good Clinical Practice” (CPMP/ICH/135/95). Participants should sign the informed consent for their inclusion in the study. The first author will individually inform the participants about the study procedure, the use of data by the authors and the laws that protect their rights subject to the “Organic Law of Data Protection”. Since our intervention does not involve any physical action, no side effects and/ or adverse reactions are expected.

This study will be conducted in accordance with the ethical principles established in Law 14/2007, of July 3, on Biomedical Research, which regulates research involving human subjects and the processing of personal data in the biomedical field. Likewise, data management will comply with Regulation (EU) 2016/679 (General Data Protection Regulation, GDPR) and Organic Law 3/2018 on the Protection of Personal Data and Guarantee of Digital Rights.

The personal data of the participants will be securely stored at the University of Lleida, which has the necessary security measures to ensure its confidentiality and integrity. These data will be retained for five years after the completion of the study, in compliance with the applicable legal requirements. The study protocol was approved by the Clinical Research Ethics Committee of Arnau de Vilanova University Hospital of Lleida (HUAV) (CEIC-3221).

## Discussion

Hundreds of millions of people worldwide are affected by neurological diseases [1,2]. The resulting dysfunction can lead to several chronic issues. Neurological diseases mainly requires life-long management [2,3].

The World Health Organization (WHO) conceptualizes disability as a universal human experience, arising from the dynamic interaction between individuals with health conditions—such as neurological disorders—and various personal and environmental factors. Such factors may involve societal perceptions, physical obstacles like inadequate transportation and public facilities, and a lack of sufficient social support [31].

Functional limitations and prolonged hospital stays can significantly impact the family dynamic, as caregivers often assume substantial and varied responsibilities over time. Effectively managing the challenges associated with caregiving is a major hurdle for these parents. Parents must juggle caregiving tasks, provide support to their child during hospitalizations and medical appointments, and make crucial decisions about treatment options [21,32]. Research suggests that families of children diagnosed with disabilities are disproportionately affected, often experiencing greater levels of instability and dysfunction compared to families of typically developing children [33].

Parenting stress is a prominent area of investigation in research examining the experiences of families with children with disabilities [34,35]. Although some families may demonstrate resiliency in the face of such stressors, the demanding treatment regimens and shifts in roles, responsibilities, and resources may negatively impact family functioning [36].

Some studies compared the parenting stress in families of children with developmental disabilities to parents of age-appropriate children, showing increased stress levels in families of children with developmental disabilities or disabilities [37–39].

Scheibner et al. [40] developed a cross-sectional study and reported that. parents of children with mental and/or physical disabilities reported the highest stress levels.

Irlbauer-Müller et al. [41] found that stressed parents had difficulty reporting their child’s behavioural disorders and problems with the child in medical interviews.

Glinac et al. [8] reported in an observational study with 411 participants, that mothers of children with Cerebral Palsy experienced a lower quality of life in all measured areas compared to mothers of healthy children. Specifically, in terms of their child’s mobility, mothers of children who were unable to move independently reported worse social functioning than mothers of children who could move on their own. Reliable Clinical Practice Guidelines (CPGs) and tools to support clinical decision-making must be developed to ensure that this population and their caregivers receive safer and more efficient care throughout the diagnostic, treatment and follow-up care process. For this purpose, it is necessary to give a voice not only to the patients themselves but also to the people who accompany them in their processes, such as their caregivers or representatives, to identify the key issues for the people affected. Early detection of underlying problems can help to offer timely and appropriate health support to prevent family malfunction [42].

The findings generated through this sequential mixed-methods strategy may offer context-specific insights, within the Spanish healthcare system, relevant for the development of clinical practice guidelines and support tools aimed at improving caregiver well-being and family functioning. Early identification of caregiver distress and unmet needs has the potential to inform timely, targeted interventions and to enhance family-centred care practices.

Overall, this protocol should be interpreted as exploratory rather than confirmatory. Its primary contribution lies in assessing the feasibility and added value of a mixed-methods approach to studying caregiver outcomes, as well as in generating hypothesis-generating, contextually grounded insights that may guide future research and service development.

### Limitations

The main limitations of this study include restricted generalizability, as it focuses on caregivers within the Spanish healthcare system, and potential self-report bias, as participants may overestimate or underestimate their stress levels and quality of life. Recruitment from a single hospital site may further limit the diversity of caregiver experiences represented, reducing external validity. However, this design ensures feasibility and methodological consistency while providing valuable context-specific insights. During the study, high caregiver workload may lead to participant dropout, affecting data collection, particularly in the qualitative phase. Discussing emotional burden may cause distress, influencing responses. Another limitation could be scheduling interviews, since it might be difficult due to caregivers’ unpredictable schedules, and response bias could arise if participants provide socially desirable rather than fully honest answers. These factors may impact data quality and the study’s overall conclusions. Future research should aim to replicate and extend the study across multiple sites to enhance generalizability.

## Supporting information

S1 Fig Participant timeline. (TIF)

S2 File SPIRIT 2025 checklist. (DOCX)

S3 File Study protocol presented to the Ethics Committee. (DOCX)
