# Supplementary figures and images for: Experiences, outcomes and unmet needs of caregivers of children with Cerebral Palsy in Spain: Protocol for a mixed-methods study
Source: PLoS One. 2026 Mar 13;21(3):e0342763. doi: 10.1371/journal.pone.0342763 (PMC12987472; doi:10.1371/journal.pone.0342763)

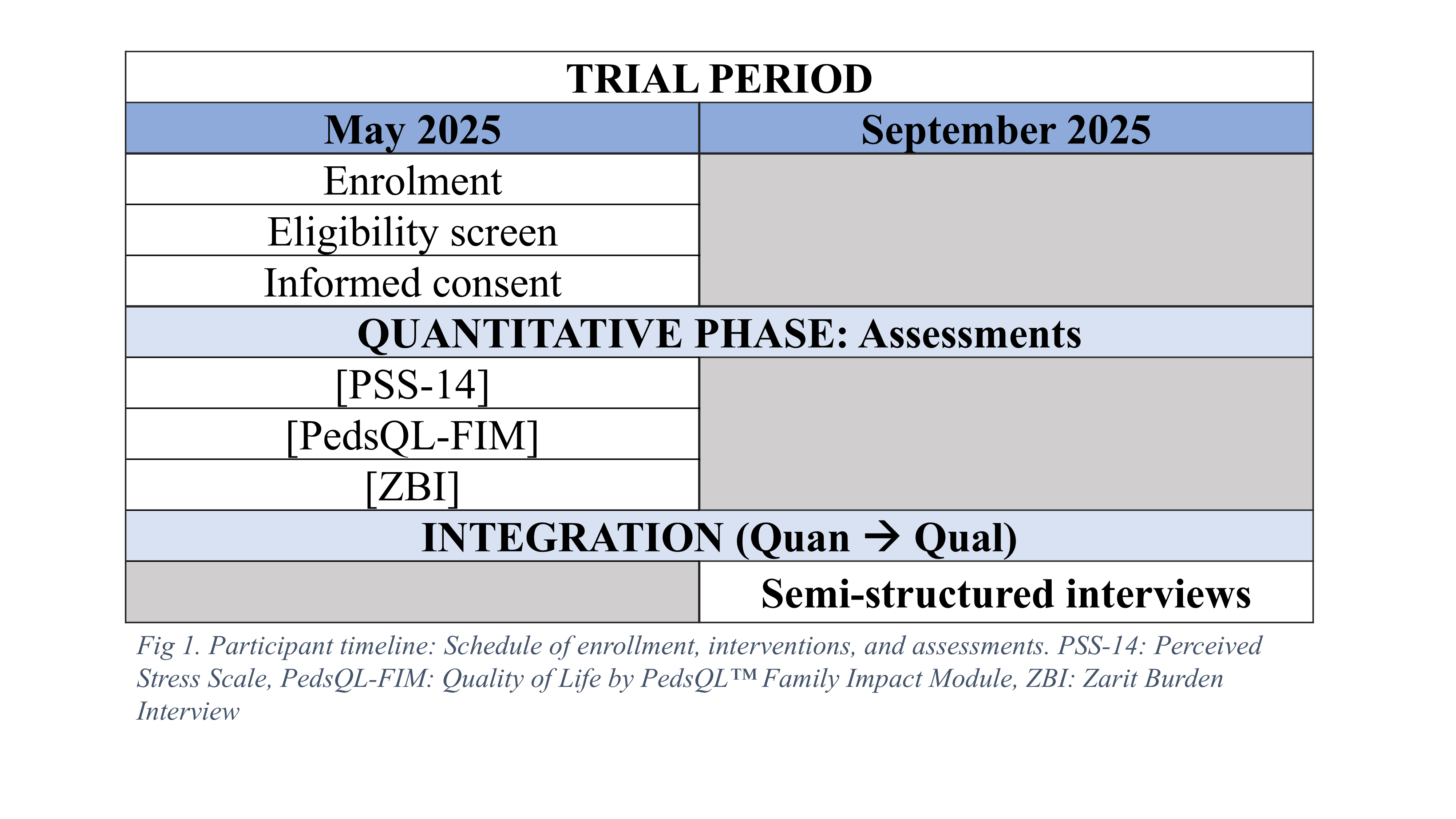

Supplement: S1 Fig — (TIF) [file pone.0342763.s001.tif]
