# Supplementary material for: Experiences, outcomes and unmet needs of caregivers of children with Cerebral Palsy in Spain: Protocol for a mixed-methods study
Source: PLoS One. 2026 Mar 13;21(3):e0342763. doi: 10.1371/journal.pone.0342763 (PMC12987472; doi:10.1371/journal.pone.0342763)
Supplement: S3 File — (DOCX) [file pone.0342763.s003.docx]

***Caregiving experiences, outcomes and unmet needs of parents or caregivers of children with Cerebral Palsy: A Mixed-Method study***

# Background

## Neurological disorders

Neurological disorders (ND) are marked by dysfunctions in various parts of the brain or nervous system (i.e. spinal cord and nerves). In other words, the brain, spinal cord, cranial nerves, peripheral nerves, nerve roots, autonomic nervous system, neuromuscular junction, and muscles. Hundreds of millions of people worldwide are affected by neurological disease (1,2). In children, these disorders can be caused by genetic conditions, neurotoxins, hypoxia, infections, and injuries. They often present as impairments in physical, memory, motor, speech, and cognitive functions. The resulting dysfunction can lead to several chronic issues. ND mainly require life-long management. Effective management of these problems necessitates a thorough understanding by physicians, caregivers, and parents (2,3).

### Disability

The World Health Organization (WHO) defines disability as part of being human and a result of the interaction between individuals with a health condition, such as neurological disorders, with personal and environmental factors including negative attitudes, inaccessible transportation and public buildings, and limited social support (4). The International Classification of Functioning, Disability and Health (ICF) is a classification of health and health-related domains (5).

Functional limitations and prolonged hospital stays can significantly impact the family dynamic, as caregivers often assume substantial and varied responsibilities over time. Effectively managing the challenges associated with caregiving is a major hurdle for these parents, which can adversely affect their physical and mental well-being and pose risks to the overall functioning of the family. Parents must juggle caregiving tasks, provide support to their child during hospitalizations and medical appointments, and make crucial decisions about treatment options (6,7) Researchers have posited that families of a child diagnosed with a disability are negatively impacted and therefore experience more instability and dysfunction than ‘‘typical’’ families (8).

Parents of children with developmental disorders (DD) or disabilities encounter unique and significant challenges in their daily lives, both in terms of caring for their child and parenting. Mothers as well as fathers, must devote considerable time and energy to adequately address and meet their child's special needs (9).

## Psychological and physical health

The psychological and physical health of caregivers, mostly mothers, wis significantly impacted by the child's behaviour and the demands of caregiving, according to Raina et al. (10) research. Behavioural issues in children are assumed to be key predictors of the caregivers' psychological well-being, influencing it both directly and indirectly by affecting their self-perception and the overall functioning of the family. Family dynamics not only seem to have a direct impact on health but also play a mediating role in how self-perception, social support, and stress management influenced well-being. Effectively managing the challenges associated with caregiving is a significant burden for parents, impacting their physical and psychological well-being and posing a risk to the overall functioning of the family (10,11).

## Parenting stress

Parenting stress is the most widely topic investigated in experience research paradigms of children with disabilities families (12,13). Although some families may demonstrate resiliency in the face of such stressors, the demanding treatment regimens and shifts in roles, responsibilities, and resources may negatively impact family functioning (14).

The birth of a child with for example, cerebral palsy, can significantly alter the family's daily life, leading parents to feel denial, sadness, and guilt. They need to adapt to their child's limitations and find ways to handle these new challenges. In this sense, the birth of a child diagnosed with a chronic illness or disability, has the potential to change the family routine, triggering feelings of denial, sadness and guilt in the parental figures who need to learn to face the limitations of their children (15,16)

Some studies compared the parenting stress in families of children with developmental disabilities or disabilities, to parents of age-appropriate children, with increased results in families of children with DD or disabilities (17–19)*.*

*Scheibner et al. (20)* developed a cross-sectional study where they compared parenting stress in families of children with various types of disabilities. Parents of children with mental and/or physical disabilities reported the highest stress levels.

In the same way, a systematic review, critically reviewed the literature on parenting stress among caregivers of children with chronic illness, reported significantly greater general parenting stress than caregivers of healthy children (14).

High levels of parenting stress and acquired mental health conditions, on the other hand, can lead to difficulties in parenting and possible child neglect and therefore negatively affect children's health and development (21). To ensure children’s optimal care and development, parenting stress must be recognised and treated as early as possible with family-centred practices (22,23).

Other investigations as *Irlbauer-Müller et al.* (24) found that stressed parents had difficulty reporting their child's behavioural disorders and problems with the child in medical interviews. The emphasis on childcare often leads to overlooking family stress factors, as they may not be directly inquired about and thus go unnoticed.

*Glinac et al.* (16) reported in an observational study with 411 participants, that mothers of children with cerebral palsy experienced a lower quality of life in all measured areas compared to mothers of healthy children. Specifically, in terms of their child's mobility, mothers of children who were unable to move independently reported worse social functioning than mothers of children who could move on their own.

Elevated levels of parenting stress and related mental health issues pose risks for damaging parent-child relationships, disrupted development, and poorer health outcomes for the child (21,25). Besides, perceived stress is associated with higher levels of anxiety and poor quality of life (26).

Reliable Clinical Practice Guidelines (CPGs) and tools to support clinical decision-making must be developed to ensure that this population and their caregivers receive safer and more efficient care throughout the diagnostic, treatment and follow-up care process. For this purpose, it is necessary to give a voice to the patients themselves and to the people who accompany them in their processes, such as their caregivers or representatives, in order to identify the key issues for the people affected. Early detection of underlying problems can help to offer timely and appropriate health support to prevent family malfunction (27).

Although qualitative research has been already made in different populations of caregivers’ experiences, as far as the authors know, there is not any qualitative study exploring emotional experiences, stress management, problems and needs of parents/caregivers of children with motor/physical disability under the umbrella of the Spanish Healthcare system. It is important to emphasize that the demands and expectations of this population may vary significantly depending on the healthcare system in each country. This distinction will help identify areas for improvement within the Spanish healthcare system.

The aim of this mixed-methods study is to investigate the caregiving experiences and outcomes of parents and caregivers of children with Cerebral Palsy, as well as to identify specific needs that could be addressed in future intervention efforts, not only technical and short-term rehabilitation interventions that are focused primarily on the child but also family centred interventions in a biopsychosocial framework.

# Methods/design

To address the research question, the authors will use an **explanatory sequential mixed-method** design consisting of three phases **(QUAN → Connection 🡪 QUAL)**. The rationale for choosing this approach is that the analysis of the quantitative data will provide a general understanding of the caregiver burden, stress and quality of life among parents of children with cerebral palsy. The subsequent analysis of the qualitative data will further explain the statistical results through an in-depth exploration of the experiences and needs of the participants (28). See flow diagram in **¡Error! No se encuentra el origen de la referencia.**.

A three-phase procedure will be used:

- QUANT phase: Quantitative data will be collected and analysed using three questionnaires, providing the authors with a comprehensive profile of participants' stress levels, quality of life, and caregiver burden. This data will also enable the identification of specific subgroups that will be the primary focus of the subsequent qualitative phase, while informing the development of new research questions for deeper exploration during this phase.
- Connection phase: Firstly, based on the categories identified in the quantitative questionnaires, the content of the semi-structured interview will be developed. Secondly, the results from the quantitative phase will help plan the qualitative phase, including the selection of a possible subsample and the development of new questions that cannot be answered with quantitative data.
- QUAL phase: semi-structured personal interviews, based on the results of the quantitative data and followed by thematic analysis.

The use of mixed-methods in this study is justified by the need to connect both quantitative and qualitative data. Integration in this study will occur at 3 points; i) The development of the qualitative interview guide will be based on the categories identified in the questionnaires ii) The results from the quantitative phase will help to plan the qualitative phase, including a potential of a subsample and the formulation of new questions iii) The qualitative data will be integrated with the quantitative data to obtain a more comprehensive understanding of the quantitative results.

## Recruitment and Data collection

### Quantitative phase

Prior to the beginning of the recruitment, the principal investigator (PI) of the study will present the project to paediatric service of the Arnau de Vilanova University Hospital of Lleida (HUAV) to ask for their cooperation. The paediatric service of HUAV will inform eligible participants about the study during their medical visits. If the patient agrees to participate, they will give their consent for the paediatric service to share their contact details with the research team. The investigators will then reach out to the participant to explain the project in detail, schedule a first meeting to sign the Informed Consent, and complete the initial questionnaires. The Informed consent will provide the first author contact in case the participants have doubts or questions about the study. Participants who signed the informed consent will also be asked to provide contact information for potential follow-up regarding the qualitative interview.

In the first meeting, the participants who comply with the inclusion criteria and signed the informed consent will be guided to a study homepage hosted on the university's website. This website will provide a link to a secure platform specifically designed for collecting research data (PedsQL-FIM, ZBI, PSS-14).

**Sociodemographic data**

These data will include descriptive information such as age, sex, household income, among others.
See Table 1.

The level of disability of the child will be based on the of the GMFCS (29). It provides a clear description of movement abilities and walking, focusing on how individuals use mobility aids, such as walkers or wheelchairs, and how they control movement in different environments.

- **Level I**: Walks without limitations. Children can walk indoors, outdoors, and climb stairs without the need for railing support. They can run and jump, although speed, balance, and coordination may be reduced.
- **Level II**: Walks with limitations. Children can walk in most settings, but they may need to use a railing to climb stairs. They have difficulty with long distances and maintaining balance on uneven surfaces. Running and jumping are limited.
- **Level III**: Walks using a hand-held mobility device. Children can walk indoors with a walker or crutches but may use a wheelchair for longer distances or outdoor mobility.
- **Level IV**: Self-mobility with limitations; may use powered mobility. Children may use a wheelchair or powered mobility device for most activities and need assistance to walk short distances or transfer between positions.
- **Level V**: Transported in a manual wheelchair. Children have severe limitations in head and trunk control, making self-mobility extremely difficult, even with assistive technology. They rely entirely on caregivers for mobility.

#### Inclusion criteria

- >18 years old
- Being a caregiver/parent of a child with Cerebral Palsy (<18y)
- Spanish/Catalan speaking
- Willing to talk about their experiences and be audio/video-recorded.
- Accept and sign the informed consent form

### Qualitative phase

The interview guide will be refined based on the results of the quantitative phase. Additionally, the findings from the quantitative questionnaires will inform the selection of participants for the interview phase. The interview will take place in the Faculty of Nursing and Physiotherapy of the University of Lleida. Interviews will be conducted in either Spanish or Catalan (participants’ choice) and will be recorded in audio with participants’ previous consent. Interview guides will be based on the categories identified at the quantitative survey, specifically based on the Spanish version of the PedsQL™ Family Impact Module (PedsQL-FIM) (30), and the Spanish version of The Zarit Burden Interview (ZBI) (31).The interviews are meant to last about an hour. See Interview Guide at Annex 6.3

### Measures

#### Perceived Stress Scale, PSS 14

To measure participants perceived stress, the Perceived Stress Scale 14 item Spanish version will be used (PSS 14). It consists of 14 items scored from 0 up to 4 (0=never, 1 = rarely 2= sometimes 3= usually 4 =almost always). The total scores on the scale can range from 0 (no stress) to 56 (extreme stress) (32).

The PSS is a short and easy to use questionnaire established with acceptable psychometric properties with the purpose of assessing the perception of stress in everyday situations over the past month. It includes questions about how often individuals experience stress-related feelings and thoughts (32,33) .

It is widely established its use in measuring the effectiveness of intervention programs such as Mindfulness-Based Stress Reduction (MBSR), cognitive-behavioural therapies, and other stress management techniques (34).

#### Quality of Life by PedsQL™ Family Impact Module

To measure the impact of paediatric chronic health conditions on parents and the family, the last developed Spanish version PedsQL™ Family Impact Module (7,35) will be used. The module measures multidimensions parent self-report; physical, emotional, social, and cognitive functioning, communication, and worry (7,36). It is used to assess health-related QoL in children aged 2-18 years. It takes up to 5-10 minutes to be completed.

It is a 36-item with an internal consistency reliability of α = 0.97 (36). It assesses 8 main factors: physical functioning (6 items), emotional functioning (5 items), social functioning (4 items), cognitive functioning (4 items), communication (3 items), worry (5 items), daily activities (3 items) and family relationships (5 items). The answers are given on a 5-point Likert scale (0 = it is never a problem, 4 = it is almost always a problem) and are reversed scored and linearly transformed to a 0-100 scale (0 = 100, 1 = 75, 2 = 50, 3 = 25, 4 = 0) so that a greater score indicates better functioning. The total score is the sum of all the 36 items divided by the number of items answered (37).

For its academic and research, it is accessible at no charge via the ePROVIDE™ Online distribution process (30). In this instance, there is no need to adapt the language of the original instrument. The research team of the Mapi Research Institute has already developed a Spanish and Catalan version of the PedsQL-FIM. The translation produced by Mapi Research Trust is validated for academic and commercial use at no charge for non-funded researchers. The authors of the present study have already acquired authorization from the Mapi Research Trust to apply this instrument.

#### Zarit Burden Interview

The Zarit Burden Interview (ZBI) is a popular caregiver self-report instrument to measure caregiver burden. ZBI assess caregiver perceptions of burden that may inadvertently affect their health, personal, social or financial wellbeing (31).

Caregivers are requested to assess the level of burden they experience while caring for a loved one. Burden is defined as the degree to which a caregiver perceives negative effects on their emotional and physical health, social life, and finances, which may hinder their ability to provide care. Responses vary from "not at all" to "extremely." It consists of 22 items; the total score is calculated by summing the scores of all the items indicated. It is suggested that from 0 to 21 means no to mild burden, from 21 to 40 means mild to moderate burden, from 41 to 60 means moderate to severe burden and more than 61 means severe burden (38).

The Spanish version developed by Martin-Carrasco et al. (39) showed a good internal reliability (Cronbach alpha coefficient of 0.92) (40,41). The questionnaire is distributed by Mapi Research Trust. It is accessible at no charge for non-funded researchers via the ePROVIDE™ Online distribution process. The authors of the present study have already acquired authorization from the Mapi Research Trust to apply this instrument (31).

**
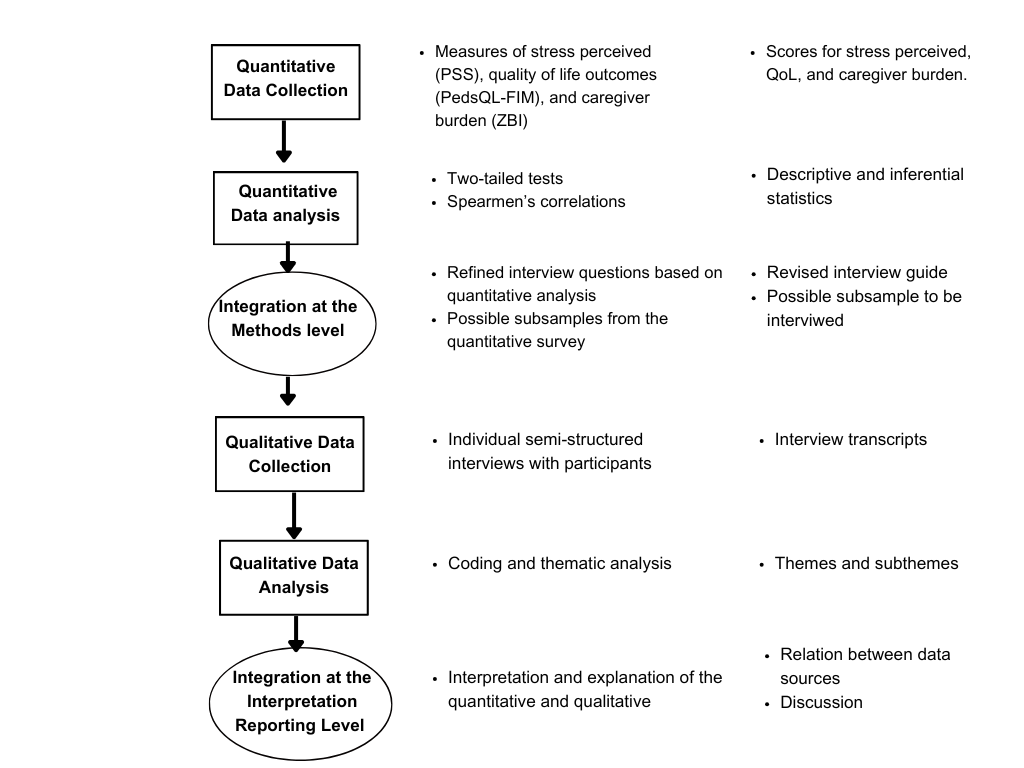
Phase Procedure Product**

Figure 1: Diagram of the study design and mixed-methods integration

## Data analysis

### Quantitative data

The quantitative data will be analysed using descriptive statistics and frequency counts. Sociodemographic characteristics will be analysed for the descriptive analysis. Descriptive statistics will be reported with means and standard deviations for continuous variables (age, child’s age, PSS, Peds-QL-FIM, ZBI) with normal distribution, or medians and interquartile ranges for variables without normal distribution. For categorical variables (sex, educational level, severity) both absolute and relative frequencies will be presented. The t-Student test will be used to compare continuous variables, while the Chi-square test will be used for categorical variables. If normal distribution cannot be assumed, a non-parametric test will be applied for bivariate analysis (e.g., questionnaires’ scores between different ages, between different household incomes, severity, etc.). Analysis will be based on 2-tailed tests with a significance level of α =.05. Statistical analysis will be performed using IBM SPSS Statistics version 28.

Additionally, Spearman’s correlations will be used to assess relationship between the different studied variables (stress, QoL and caregiver burden).

### Qualitative data

Before starting the qualitative analysis, the interviews will be transcribed word-for-word and reviewed by the authors. Both deductive and inductive (free or emerging) coding methods will be applied to identify and integrate the data, leading to the creation of various meaning categories (i.e., sentences or paragraphs with the same meaning). These categories will be used to generate conceptual themes. The approach will follow the Consolidated Criteria for Reporting Qualitative Research. This approach allows for analysis of the data with a set of expected themes informed by previous knowledge or research (42).

The first author will handle the interview analysis, while a different researcher will be involved in information triangulation to ensure the validity and reliability of the materials (43). Subsequently, all authors will contribute to interpreting the data. The ATLAS.ti software will be used for coding and managing the information (44,45).

# Ethical issues

The study follows the Declaration of Helsinki and the “Guidelines for Good Clinical Practice” (CPMP/ICH/135/95). Participants should sign the informed consent for their inclusion in the study. The first author will individually inform the participants about the study procedure, the use of data by the authors and the laws that protect their rights subject to the “Organic Law of Data Protection”. Since our intervention does not involve any physical action, no side effects and / or adverse reactions are expected.

This study will be conducted in accordance with the ethical principles established in Law 14/2007, of July 3, on Biomedical Research, which regulates research involving human subjects and the processing of personal data in the biomedical field. Likewise, data management will comply with Regulation (EU) 2016/679 (General Data Protection Regulation, GDPR) and Organic Law 3/2018 on the Protection of Personal Data and Guarantee of Digital Rights.

The personal data of the participants will be securely stored at the University of Lleida, which has the necessary security measures to ensure its confidentiality and integrity. These data will be retained for five years after the completion of the study, in compliance with the applicable legal requirements.

All participants will receive detailed information regarding the study’s objectives, the use of their personal data, and their rights. Before participating, they will sign the informed consent form, thus ensuring their voluntary and informed participation.

# Validity and reliability/rigour

The description of the project's mixed methodology was developed based on the recommendations of the Good Reporting of a Mixed Methods Study (GRAMMS) statement (46). The Consolidated Criteria for Reporting Qualitative Research (47) were also considered to report the qualitative methodology and the Consolidated Standards of Reporting Trials (CONSORT 2010) guidelines to report the quantitative methodology (48).

# Bibliography

1. Mental health: neurological disorders [Internet]. [cited 2024 Jul 26]. Available from: https://www.who.int/news-room/questions-and-answers/item/mental-health-neurological-disorders

2. Banerjee TK, Hazra A, Biswas A, Ray J, Roy T, Raut DK, et al. Neurological disorders in children and adolescents. Indian J Pediatr [Internet]. 2009 Feb 4 [cited 2024 Jul 21];76(2):139–46. Available from: https://link.springer.com/article/10.1007/s12098-008-0226-z

3. What is a Neurologic Disorder? - Child Neurology Foundation [Internet]. [cited 2024 Jul 26]. Available from: https://www.childneurologyfoundation.org/what-is-a-neurologic-disorder/

4. Disability [Internet]. [cited 2024 Jul 21]. Available from: https://www.who.int/health-topics/disability#tab=tab_1

5. International Classification of Functioning, Disability and Health (ICF) [Internet]. [cited 2024 Jul 21]. Available from: https://www.who.int/standards/classifications/international-classification-of-functioning-disability-and-health

6. Piran P, Khademi Z, Tayari N, Mansouri N. Caregiving burden of children with chronic diseases. Electron Physician [Internet]. 2017 Sep 25 [cited 2024 Sep 24];9(9):5380–7. Available from: https://pubmed.ncbi.nlm.nih.gov/29038725/

7. Ortega J, Vázquez N, Amayra Caro I, Assalone F. Psychometric properties of the Spanish version of the Pediatric Quality of Life Inventory Family Impact Module (PedsQL FIM). Anales de Pediatría (English Edition). 2023 Jan 1;98(1):48–57.

8. Watson SL, Hayes SA, Radford-Paz E. “Diagnose me Please!”: A Review of Research about the Journey and Initial Impact of Parents Seeking a Diagnosis of Developmental Disability for their Child. Int Rev Res Dev Disabil. 2011 Jan 1;41(C):31–71.

9. Bhopti A, Brown T, Lentin P. Family Quality of Life. http://dx.doi.org/101177/1053815116673182 [Internet]. 2016 Oct 18 [cited 2024 Jul 21];38(4):191–211. Available from: https://journals.sagepub.com/doi/10.1177/1053815116673182

10. Raina P, O’Donnell M, Rosenbaum P, Brehaut J, Walter SD, Russell D, et al. The health and well-being of caregivers of children with cerebral palsy. Pediatrics [Internet]. 2005 Jun [cited 2024 Sep 24];115(6). Available from: https://pubmed.ncbi.nlm.nih.gov/15930188/

11. Jalil YF, Villarroel GS, Silva AA, Briceño LS, Ormeño VP, Ibáñez NS, et al. Reliability and validity of the revised impact on family scale (RIOFS) in the hospital context. J Patient Rep Outcomes [Internet]. 2019 Dec 1 [cited 2024 Sep 24];3(1). Available from: /pmc/articles/PMC6517451/

12. Davis NO, Carter AS. Parenting stress in mothers and fathers of toddlers with autism spectrum disorders: associations with child characteristics. J Autism Dev Disord [Internet]. 2008 Aug [cited 2024 Jul 18];38(7):1278–91. Available from: https://pubmed.ncbi.nlm.nih.gov/18240012/

13. Pisula E. A comparative study of stress profiles in mothers of children with autism and those of children with down’s syndrome. Journal of Applied Research in Intellectual Disabilities. 2007 May;20(3):274–8.

14. Cousino MK, Hazen RA. Parenting Stress Among Caregivers of Children With Chronic Illness: A Systematic Review. [cited 2024 Jul 18]; Available from: https://academic.oup.com/jpepsy/article/38/8/809/919245

15. Cunha K da C, Pontes FAR, Silva SS da C, Cunha K da C, Pontes FAR, Silva SS da C. Parenting Stress and Motor Function of Children with Cerebral Palsy. Psychology [Internet]. 2016 Dec 30 [cited 2024 Jul 26];8(1):44–58. Available from: http://www.scirp.org/journal/PaperInformation.aspx?PaperID=73287

16. Glinac A, Matović L, Delalić A, Mešalić L. Quality of Life in Mothers of Children with Cerebral Palsy. Acta Clin Croat [Internet]. 2017 [cited 2024 Jul 26];56(2):299–307. Available from: https://pubmed.ncbi.nlm.nih.gov/29485798/

17. Baker BL, McIntyre LL, Blacher J, Crnic K, Edelbrock C, Low C. Pre-school children with and without developmental delay: behaviour problems and parenting stress over time. Journal of Intellectual Disability Research [Internet]. 2003 May 1 [cited 2024 Jul 18];47(4–5):217–30. Available from: https://onlinelibrary.wiley.com/doi/full/10.1046/j.1365-2788.2003.00484.x

18. Huang YP, Chang MY, Chi YL, Lai FC. Health-related quality of life in fathers of children with or without developmental disability: The mediating effect of parental stress. Quality of Life Research [Internet]. 2014 Feb 10 [cited 2024 Jul 18];23(1):175–83. Available from: https://link.springer.com/article/10.1007/s11136-013-0469-7

19. Barak-Levy Y, Atzaba-Poria N. A mediation model of parental stress, parenting, and risk factors in families having children with mild intellectual disability. Res Dev Disabil. 2020 Mar 1;98:103577.

20. Scheibner C, Scheibner M, Hornemann F, Arélin M, Hennig YD, Kiep H, et al. Parenting stress in families of children with disabilities: Impact of type of disability and assessment of attending paediatricians. Child Care Health Dev. 2024 Jan 1;50(1).

21. Éthier LS, Lacharité C, Couture G. Childhood adversity, parental stress, and depression of negligent mothers. Child Abuse Negl. 1995 May 1;19(5):619–32.

22. Dunst CJ. Placing Parent Education in Conceptual and Empirical Context. Topics Early Child Spec Educ. 1999;19(3):141–7.

23. Bailey DB, Nelson L, Hebbeler K, Spiker D. Modeling the impact of formal and informal supports for young children with disabilities and their families. Pediatrics [Internet]. 2007 Oct [cited 2024 Jul 18];120(4). Available from: https://pubmed.ncbi.nlm.nih.gov/17893190/

24. Irlbauer-Müller V, Eichler A, Stemmler M, Moll GH, Kratz O. Elterliche Belastung und die Zuverlässigkeit von Elternangaben                    in der Diagnostik psychisch und verhaltensauffälliger Kinder und                    Jugendlicher. https://doi.org/101024/1422-4917/a000467 [Internet]. 2016 Aug 18 [cited 2024 Jul 18];45(4):303–9. Available from: https://econtent.hogrefe.com/doi/10.1024/1422-4917/a000467

25. Bourke-Taylor HM, Joyce KS, Grzegorczyn S, Tirlea L. Profile of Mothers of Children with a Disability Who Seek Support for Mental Health and Wellbeing. J Autism Dev Disord [Internet]. 2022 Sep 1 [cited 2024 Jul 18];52(9):3800–13. Available from: https://link.springer.com/article/10.1007/s10803-021-05260-w

26. Racic M, Todorovic R, Ivkovic N, Masic S, Joksimovic B, Kulic M. Self-perceived stress in relation to anxiety, depression and health-related quality of life among health professions students: A cross-sectional study from Bosnia and Herzegovina. Zdr Varst. 2017;56(4):251–9.

27. Wittenberg E, Saada A, Prosser LA. How illness affects family members: a qualitative interview survey. Patient [Internet]. 2013 Dec [cited 2024 Sep 24];6(4):257–68. Available from: https://pubmed.ncbi.nlm.nih.gov/24142495/

28. Ivankova N V., Creswell JW, Stick SL. Using Mixed-Methods Sequential Explanatory Design: From Theory to Practice. http://dx.doi.org.utrechtuniversity.idm.oclc.org/101177/1525822X05282260 [Internet]. 2006 Feb 1 [cited 2024 Sep 22];18(1):3–20. Available from: https://journals-sagepub-com.utrechtuniversity.idm.oclc.org/doi/10.1177/1525822X05282260

29. CanChild [Internet]. [cited 2024 Sep 18]. Available from: https://canchild.ca/en/resources/42-gross-motor-function-classification-system-expanded-revised-gmfcs-e-r

30. Official PedsQLTM | Pediatric Quality of Life InventoryTM distributed by Mapi Research Trust | ePROVIDE [Internet]. [cited 2024 Jul 19]. Available from: https://eprovide.mapi-trust.org/instruments/pediatric-quality-of-life-inventory

31. Official ZBI | Zarit Burden Interview distributed by Mapi Research Trust | ePROVIDE [Internet]. [cited 2024 Oct 28]. Available from: https://eprovide.mapi-trust.org/instruments/zarit-burden-interview

32. Cohen S, Kamarck T, Mermelstein R. A global measure of perceived stress. J Health Soc Behav. 1983;24(4):385–96.

33. Lee EH. Review of the Psychometric Evidence of the Perceived Stress Scale. Asian Nurs Res (Korean Soc Nurs Sci) [Internet]. 2012 [cited 2024 Jul 28];6:121–7. Available from: http://dx.doi.org/10.1016/j.anr.2012.08.004

34. Grossman P, Niemann L, Schmidt S, Walach H. Mindfulness-based stress reduction and health benefits: A meta-analysis. J Psychosom Res. 2004 Jul 1;57(1):35–43.

35. PedsQL TM (Pediatric Quality of Life Inventory TM) [Internet]. [cited 2024 Jul 19]. Available from: https://www.pedsql.org/about_pedsql.html

36. Varni JW, Sherman SA, Burwinkle TM, Dickinson PE, Dixon P. The PedsQLTM Family Impact Module: Preliminary reliability and validity. Health Qual Life Outcomes [Internet]. 2004 Sep 27 [cited 2024 Jul 19];2:55. Available from: /pmc/articles/PMC521692/

37. SCALING AND SCORING for the Acute and Standard versions OF THE. [cited 2024 Jul 19]; Available from: https://eprovide.mapi-trust.org/

38. Zarit Burden Interview Assessing Caregiver Burden.

39. Martín Carrasco M. Adaptación para nuestro medio de la Escala de Sobrecarga del Cuidador de Zarit. Revista multidisciplinar de gerontología, ISSN 1139-0921, Vol 6, No 4, 1996, pág 338 [Internet]. 1996 [cited 2024 Sep 25];6(4):338. Available from: https://dialnet.unirioja.es/servlet/articulo?codigo=2959659

40. Martin-Carrasco M, Otermin P, Perez-Camo V, Pujol J, Aguera L, Martin MJ, et al. EDUCA study: Psychometric properties of the Spanish version of the Zarit Caregiver Burden Scale. Aging Ment Health [Internet]. 2010 Aug [cited 2024 Sep 25];14(6):705–11. Available from: https://pubmed.ncbi.nlm.nih.gov/20544413/

41. Isabel Casado Morales Ma Xesús Froján Parga Ma Eugenia Olivares Crespo Miguel Ángel Pérez Nieto Viente Prieto Cabras Ma Fe Rodríguez Muñoz Pablo Santamaría Fernández Albert Sesé Abad M, Crespo M, Teresa Rivas M. La evaluación de la carga del cuidador: una revisión más allá de la escala de Zarit. https://journals.copmadrid.org/clysa [Internet]. 2015 Feb 23 [cited 2024 Sep 25];26(1):9–15. Available from: https://journals.copmadrid.org/clysa/art/j.clysa.2014.07.002

42. Tong A, Sainsbury P, Craig J. Consolidated criteria for reporting qualitative research (COREQ): a 32-item checklist for interviews and focus groups. International Journal for Quality in Health Care [Internet]. 2007 Dec 1 [cited 2024 Sep 18];19(6):349–57. Available from: https://dx-doi-org.utrechtuniversity.idm.oclc.org/10.1093/intqhc/mzm042

43. Vaismoradi M, Turunen H, Bondas T. Content analysis and thematic analysis: Implications for conducting a qualitative descriptive study. Nurs Health Sci [Internet]. 2013 Sep 1 [cited 2024 Sep 16];15(3):398–405. Available from: https://onlinelibrary.wiley.com/doi/full/10.1111/nhs.12048

44. Graneheim UH, Lundman B. Qualitative content analysis in nursing research: Concepts, procedures and measures to achieve trustworthiness. Nurse Educ Today [Internet]. 2004 [cited 2024 Sep 16];24(2):105–12. Available from: https://pubmed.ncbi.nlm.nih.gov/14769454/

45. ATLAS.ti | The #1 Software for Qualitative Data Analysis - ATLAS.ti [Internet]. [cited 2024 Sep 18]. Available from: https://atlasti.com/

46. O’Cathain A, Murphy E, Nicholl J. The quality of mixed methods studies in health services research. J Health Serv Res Policy [Internet]. 2008 Apr [cited 2024 Sep 16];13(2):92–8. Available from: https://pubmed.ncbi.nlm.nih.gov/18416914/

47. Tong A, Sainsbury P, Craig J. Consolidated criteria for reporting qualitative research (COREQ): a 32-item checklist for interviews and focus groups. International Journal for Quality in Health Care [Internet]. 2007 Dec 1 [cited 2024 Sep 16];19(6):349–57. Available from: https://dx.doi.org/10.1093/intqhc/mzm042

48. Moher D, Hopewell S, Schulz KF, Montori V, Gøtzsche PC, Devereaux PJ, et al. CONSORT 2010 explanation and elaboration: updated guidelines for reporting parallel group randomised trials. Int J Surg [Internet]. 2012 [cited 2024 Sep 16];10(1):28–55. Available from: https://pubmed.ncbi.nlm.nih.gov/22036893/

# ANNEX

## Annex 1: Informed Consent

INFORMED CONSENT

## Annex 2: Sociodemographic data

| ***Sociodemographic  category*** | | ***Overall, n*** | ***Overall (%)*** |
| --- | --- | --- | --- |
| ***Sex*** | | | |
| *Male* | |  | |
| *Female* | |  |  |
| *Do not want to specify* | |  |  |
| ***Age*** | | | |
| *18-30* | |  | |
| *31-50* | |  |  |
| *>50* | |  |  |
| ***Diagnosed pathologies*** | *Specify/none* | |  |
|  |  | |  |
| ***Medication*** | *Specify/none* | |  |
|  |  | |  |
| ***Supplements (not medication)*** | *Specify/none* | |  |
|  |  | |  |
| ***Educational level*** | |  | |
| *Primary school* | |  | |
| *Secondary school* | |  |  |
| *High level studies* | |  |  |
| ***Relationship with the child*** | |  | |
| *Father* | |  | |
| *Mother* | |  | |
| *Caregiver* | |  | |
| ***Family members*** | |  | |
| *Single-parent* | |  | |
| *Dual-parent* | |  | |
| ***Household income*** *(single/dual)* | |  | |
| *<24.000 € / 12.000*  *24.000€ > X < 60.000€*  *12.000€ > X < 30.000*  *>60.000€ /30.000 €* | |  | |
| ***Child’s age*** | |  | |
| *<6* | |  | |
| *7-12* | |  |  |
| *13-18* | |  |  |
| ***GMFCS*** | |  | |
| *Level 1* | |  | |
| *Level 2* | |  |  |
| *Level 3* | |  |  |
| *Level 4* | |  |  |
| *Level 5* | |  |  |
| ***Type of CP*** | |  | |
| *Spastic* | |  | |
| *Hemiplegia* | |  | |
| *Diplegia* | |  | |
| *Monoplegia* | |  | |
| *Tetraplegia* | |  | |
| *Dystonic*  *Ataxic*  *Mixed* | |  | |

*Table 1 Sociodemographic characteristics*

## Annex 3: Interview guide

**Interview guide adapted into Spanish language**

1. Función física

a. ¿Cómo ha afectado tu salud física el cuidado de tu hijo/a?

b. ¿Te sientes con menos energía para tus actividades diarias? ¿Cuáles específicamente?

c. ¿Has tenido dolor físico relacionado con el cuidado de tu hijo/a?

1. Función emocional
   1. ¿Cómo te sientes emocionalmente al cuidar a tu hijo/a?
   2. ¿Cómo describirías tu estado emocional actual?
   3. ¿Cómo gestionas las emociones difíciles relacionadas con el cuidado de tu hijo/a?
2. Relaciones sociales
   1. ¿Cómo han cambiado tus relaciones sociales desde que cuidas a tu hijo/a?
   2. ¿Consideras que tus relaciones y actividades sociales son diferentes a las de otros padres?
   3. ¿Qué actividades te gustaría realizar, pero no puedes debido a las necesidades de tu hijo/a?
3. Función cognitiva
   1. ¿Has notado cambios en tu memoria, concentración u otras capacidades mentales desde que cuidas a tu hijo/a?
4. Comunicación
   1. ¿Sientes que los que te rodean comprenden tu situación?
   2. ¿Te resulta difícil expresar tus sentimientos o necesidades al personal médico?
5. Preocupaciones
   1. ¿Cuáles son tus principales preocupaciones actuales con relación a tu hijo/a, a ti mismo/a y al futuro?
   2. ¿Crees que tienes diferentes preocupaciones que los padres de niños/as sin discapacidad? ¿En qué sentido?
6. Relación familiar
   1. Si tienes pareja, ¿Cómo describirías la relación y la comunicación entre ambos?
   2. ¿Cómo es la relación con el resto de la familia?
7. Situación económica
   1. Tanto si tienes pareja como si no, ¿De qué manera se ha visto afectada vuestra/tu situación económica?
8. Necesidades
   1. ¿Qué recursos o apoyos te serían más útiles para cuidar de tu hijo/a?
   2. ¿Has recibido algún tipo de recurso o apoyo para el cuidado de tu hijo/a anteriormente? En caso de ser afirmativo, ¿Cuáles te han sido de mayor ayuda?
   3. ¿Crees que el sistema de salud podría hacer algo más por ayudaros en esta situación?
   4. ¿Qué temas o problemas relacionados con la parálisis cerebral infantil crees que deberían ser prioridad para la investigación científica?
9. Ámbito escolar
   1. ¿Qué elementos del sistema escolar crees que favorecen al aprendizaje de tu hijo/a? ¿Y cuáles lo dificultan?
   2. ¿Crees que tu hijo/a recibe la atención adecuada en la escuela? ¿Por qué?
   3. ¿Qué recursos o cambios crees que serían beneficiosos para tu hijo/a en su entorno escolar?
   4. ¿Cuáles son tus esperanzas o metas para la educación de tu hijo/a en el futuro?
